# Identification of immune signatures in Parkinson’s disease based on co-expression networks

**DOI:** 10.3389/fgene.2023.1090382

**Published:** 2023-01-17

**Authors:** Xiaolin Dong, Yanping Li, Qingyun Li, Wenhao Li, Gang Wu

**Affiliations:** Department of Neurology, The Affiliated Yan’An Hospital of Kunming Medical University, Kunming, Yunnan, China

**Keywords:** Parkinon’s disease, bioinformatics analysis, co-expression network, immunity, diagnostic markers

## Abstract

Parkinson’s disease (PD) is a common neurodegenerative disease in middle-aged and elderly people, and there is less research on the relationship between immunity and PD. In this study, the protein-protein interaction networks (PPI) data, 2747 human immune-related genes (HIRGs), 2078 PD-related genes (PDRGs), and PD-related datasets (GSE49036 and GSE20292) were downloaded from the Human Protein Reference Database (HPRD), Amigo 2, DisGeNET, and Gene Expression Omnibus (GEO) databases, respectively. An immune- or PD-directed neighbor co-expressed network construction (IOPDNC) was drawn based on the GSE49036 dataset and HPRD database. Furthermore, a PD-directed neighbor co-expressed network was constructed. Modular clustering analysis was performed on the genes of the gene interaction network obtained in the first step to obtain the central core genes using the GraphWeb online website. The modules with the top 5 functional scores and the number of core genes greater than six were selected as PD-related gene modules. The Gene Ontology (GO) and Kyoto Encyclopedia of Genes and Genomes (KEGG) enrichment analyses of different module genes were performed. The single sample Gene Set Enrichment Analysis (ssGSEA) algorithm was used to calculate the immune cell infiltration of the PD and the normal samples. The quantitative Reverse Transcription Polymerase Chain Reaction (qRT-PCR) was performed to investigate the expression of module genes. An IOPDNC and PD-directed neighbor co-expressed network (PDNC network) were constructed. Furthermore, a total of 5 immune-PD modules were identified which could distinguish between PD and normal samples, and these module genes were strongly related to PD in protein interaction level or gene expression level. In addition, functional analysis indicated that module genes were involved in various neurodegenerative diseases, such as Alzheimer disease, Huntington disease, Parkinson disease, and Long-term depression. In addition, the genes of the 6 modules were significantly associated with these 4 differential immune cells (aDC cells, eosinophils, neutrophils, and Th2 cells). Finally, the result of qRT-PCR manifested that the expression of 6 module genes was significantly higher in normal samples than in PD samples. In our study, the immune-related genes were found to be strongly related to PD and might play key roles in PD.

## Introduction

PD is the second most common neurodegenerative disease after Alzheimer’s disease (Del Rey et al., 2018). According to the report, the incidence rate is about 1%–2% (Tarsy, 2012) in the elderly over 60. The typical symptoms are static tremor, slow movement, increased muscle tone, abnormal postural gait, and some non-motor symptoms such as insomnia and constipation. The main pathological manifestations of the disease are degeneration and loss of nigra dopaminergic neurons and abnormal accumulation of α-synuclein (α-syn) (Kalia and Lang, 2016). However, the occurrence of disease also involves the influence of the environment and epigenetics, so additional research on these underlying factors is required (Elsworth, 2020).

By analyzing the mRNA expression levels of inflammatory mediators, it was found that the intensity of inflammation in PD nigra was notably increased (Pajares et al., 2020). Neuroinflammatory markers include reactive CNS myeloid cells, T lymphocytes, and increased proinflammatory cytokines/chemokines in the blood, cerebrospinal fluid (CSF), and brain parenchyma of the patients (Marras et al., 2018). These inflammatory markers change with elevated levels of T cells and autoantibodies (anti-α-syn and anti-GM1-gangliosides) in peripheral blood and CSF of PD patients. The accumulation of α-syn triggers an immune response characterized by inflammation (Kline et al., 2021). In rat studies, overexpression of α-syn was found to cause microglial activation and release of inflammatory factors (IFN-γ and resolvin D1) (Krashia et al., 2019). Moreover, α-syn can trigger neuronal autoantigen presentation (Cebrián et al., 2014), which relies on MHC I and MHC II. There are a large number of drugs that have been proven to be effective in the treatment of PD. These drugs mainly include anti-melanin antibodies (Double et al., 2009), α-syn-related drugs (Yanamandra et al., 2011; Horvath et al., 2017; Huang et al., 2019), and GM1 ganglioside-related immune responsers (Zappia et al., 2002). All these studies suggest that the pathogenesis and progression of PD may be related to the immune response.

In recent years, with the development of bioinformatics analysis, many significant advances have been made in a wide range of diseases. Several potentially therapeutic drugs (Sun et al., 2016) and key pathways (Zhang et al., 2012) have been identified by bioinformatics in PD. As the genes and the proteins they encode play key roles in physiological activities, it would be useful to study their networks in the disease. In our study, the association between immunity and PD was systematically analyzed using bioinformatics techniques based on the construction of co-expression network, providing a new perspective for the treatment and research in PD.

## Materials and methods

### Data source

The high‐confidence protein‐protein interaction (PPI) data with score >10000 were downloaded from the Human Protein Reference Database (HPRD, http://www.hprd.org/). The 2747 immune-related genes (HIRGs) were downloaded from the Amigo 2 database (http://amigo.geneontology.org/amigo) with immune as the key word. The 2078 PD-related genes (PDRGs) were downloaded from the DisGeNET database (https://www.disgenet.org/search). The FPKM expression profiles of GSE49036 and GSE20292 datasets were downloaded from the Gene Expression Omnibus (GEO) database. In the GSE49036 dataset, 8 normal and 14 PD samples were selected for data analysis, and 15 normal and 11 PD samples of GSE20292 dataset were selected for validation analysis. The clinical characteristics of GSE20292 and GSE49036 datasets were shown in Supplementary Table S1.

### Construction of an immune- or PD-directed neighbor co-expressed network construction (IOPDNC)

The fragments per kilobase of transcript per million fragments mapped (FPKM) values of gene expression in the GSE49036 dataset were log2-transformed, and the Pearson correlation of the two genes was calculated using the R package psych (version 2.1.9). Then, according to a threshold of |Pearson coefficient value| > 0.7 and FDR <0.05 to obtain the correlation among genes. Furthermore, based on the correlation between the filtered genes, mapped into the protein interaction network of the HPRD database, the common network was selected. The common network was drawn using Cytoscape software (version 3.8.2) (Shannon et al., 2003). According to the high connectivity score of genes in the common network, the number of four types of genes (PD, immune-PD, immune, and others) was counted. The Veen online tool (http://www.bioinformatics.com.cn/static/others/jvenn/example.html) was used to draw a Venn diagram of the correlation of protein-interacting genes, PDRGs, and immune genes. The R package ggplot (version 2.3.3.2) (Villanueva, 2019) was applied to draw a histogram of the four gene categories.

### Dissecting PD and immune-associated gene features in network

Based on the above-mentioned high connectivity score of genes in the common network, the core genes of PD-related genes were extracted, including PD and immune-PD genes, and their connected genes. Next, only the PD genes and their direct-acting genes were extracted as core genes. Subsequently, the number of four types of genes (PD, immune-PD, immune, other) were counted, and visualized by ggplot (Version 2.3.3.2) package (Villanueva, 2019). Notably, the immune-PD genes were both immune-related genes and PD-related genes. In the interaction network where the core gene was only PD gene, the correlation of different types of genes was calculated. Finally, the expression data of all genes in the network (the core genes are only the PD genes) were extracted and compared with the core genes. Wilcoxon rank-sum test was applied to compare the coexpression correlation coefficients between different gene groups (immune, immune-PD, PD, and other genes). Subsequently, to investigate the level of interaction between different gene groups with neighbors, cumulative distribution function (CDF) was utilized to assess the degree of the expression correlation for each gene group. The Pearson correlations of genes and the genes that related to the corresponding core genes were calculated, and the R package pheatmap was used to draw correlation heatmaps.

### Module cluster analysis and validation of its classification power

Modular clustering analysis was performed on the genes in the IOPDNC to obtain the central core genes using the GraphWeb online website (https://biit.cs.ut.ee/graphweb/). Subsequently, the number of core genes (i.e. PD-related genes) was adjusted to six in the GraphWeb database, with the rest set to default, and the modules with top five functional score values were selected as PD-related gene modules. Then, the R package ConsensusClusterPlus (version 1.54.0) (Wilkerson and Hayes, 2010) was used to perform consistent clustering analysis on the genes with the top 5 functional scores in the module, and the appropriate K value was selected based on the clustering results. In the external validation set GSE20292 dataset, the expression levels of modular genes were extracted in the same way and the accuracy of our screening of modular genes was validated against the same consistent clustering criteria.

### Functional enrichment analysis and pathway enrichment analysis of modular genes

The Gene Ontology (GO) and Kyoto Encyclopedia of Genes and Genomes (KEGG) pathway enrichment analyses of different module genes were analyzed using the R package Clusterprofiler package (version 4.0.2) (Wu et al., 2021). According to the significance threshold *p* < 0.05 and count value, the enrichment analysis of each module was carried out separately, and the ggplot (version 2.3.3.2) was used for plotting. According to the website of Pathview (https://pathview.uncc.edu/), the immune-related pathway hsa04650 was selected to visualize the most immune-related pathways in the module.

### Differential analysis of immune cell infiltration by modular genes

Based on the 24 immune cell sets, the single sample Gene Set Enrichment Analysis (ssGSEA) algorithm was used to calculate the immune cell infiltration of the PD and normal samples, and the rank sum test was used to analyze the immune differences between the PD and normal samples of cell infiltration.

### Blood samples correlation

Peripheral blood mononuclear cells (PBMC) samples from eight normal samples and eight PD patients was collected using vacuum blood tubes containing EDTA anticoagulant in accordance with clinical blood collection techniques. Each PBMC sample was gently shaken repeatedly and loaded into a 4°C thermostat and transferred to the laboratory for subsequent manipulation according to biosafety requirements.

### The quantitative reverse transcription polymerase chain reaction (qRT-PCR) analysis

The total RNA of 16 PBMC samples (8 normal samples and 8 PD samples) was extracted to verify the results of the bioinformatics analysis. The top 1 gene of each module (module 1, module 2, module 3, module 4, module 5, and module all) was selected for qRT-PCR experiments. The total RNA of 16 samples was extracted with TRIzol Reagent (Life Technologies-Invitrogen, Carlsbad, CA, United States). Then, these total RNA were reverse transcription into cDNA with the SureScript-First-strand-cDNA-synthesis-kit (Genecopoeia, Guangzhou, China) prior to qRT-PCR. The primers of these genes for qPCR were as follows:

PSMB7-For:CATGGGTTCTGGCTCCTTGG; PSMB7-Rev:CTGGTCCCCTTCTTGTTGGG; GRIN1-For:CAAGAAGGAGTGGAATGGGATG; GRIN1-Rev:GCTCGTTGTT TATGGTTAGCGG; NME1-For:CAACCCTGCAGACTCCAAGC; NME1-Rev:GGTGAAACCACAAGCCGATC; SIN3B-For:ACCCTGCCACCTACAACGG; SIN3B-Rev:TTGTCAGAGGCGAC TGTATGTTTA; HABP4-For:GAGGCAGGCAGACTTCACAG HABP4-Rev:CGAACTCCACATCCACCCAT; STX1A-For:CAATGTGGAACACGCGGTAG; STX1A-Rev: ACA​GTG​GAG​GCG​ATG​ACG​AT.

The expression was uniformized to the internal reference GAPDH and computed employing the 2^−ΔΔCt^ method.

### Western blotting

RIPA Lysis Bufferb (Servucebui) containing a protease inhibitor (Servucebio) was utilized to obtain the protein from tissues, subsequently, immunoblotting was performed. The bicinchoninic acid (BCA) quantification kit was applied to determine protein concentration of the cell lysates. The protein samples were loaded and separated by SDS-PAGE and shifted to PVDF membranes (Millpore, Sigma). Membranes were incubated with specific primary antibodies against PSMB7 (cst), GRIN1 (Affinity), NME1 (Affinity), SIN3B (Affinity), HABP4 (Proteintech), STX1A (BOSTER), and β-Actin (Proteintech) after blocked with 5% nonfat dry milk at 4 °C. Furthermore, secondary antibodies (IgG) were incubated at room temperature for 60 min and visualized using an ECL system. The dilution factors of the primary and secondary antibodies were shown in Supplementary Table S2.

## Results

### The construction of IOPDNC

**FIGURE 1:**
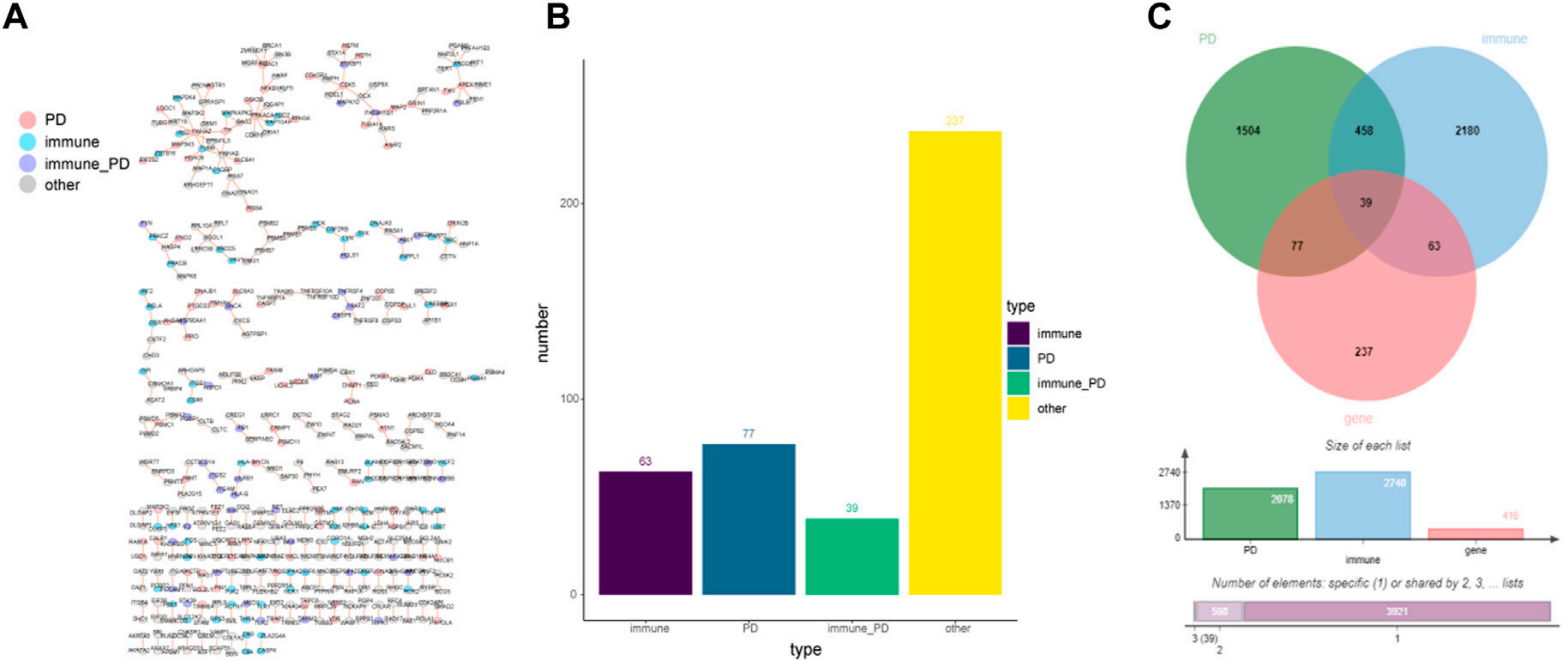
The immune- or PD-directed neighbor co-expressed network (IOPDNC network). **(A)** The global IOPDNC network was constructed to identify a common network by the GSE49036 dataset and HPRD database. **(B)** The histogram chart of the common genes in the IOPDNC network. **(C)** The Venn diagram showed the intersections of immune-related genes, PD-related genes, and common network genes.

A total of 1022077 gene relationship pairs were selected from the GSE49036 dataset. Then, the common network with 416 nodes and 281 edges was obtained from the GSE49036 dataset and HPRD database (Figure 1A, Supplementary Table S3). Among the common network genes of Figure 1A, the number of immune genes was 63; the number of PD genes was 77; the number of immune-PD genes was 39; the number of other genes were 237 (Figure 1B). Genes with high protein interaction connectivity scores were selected, and a Venn diagram of protein-interacting genes associated with PD and immune genes was drawn. There were 39 intersection genes between 2078 PD genes, 2740 immune genes, and 416 common network genes (Figure 1C). The results indicated that immune‐related genes played a vital role in the IOPDNC network. Together, these results suggested that immune-related genes might be important contributors for PD.

### Dissecting PD and immune-associated gene features in the network

**FIGURE 2:**
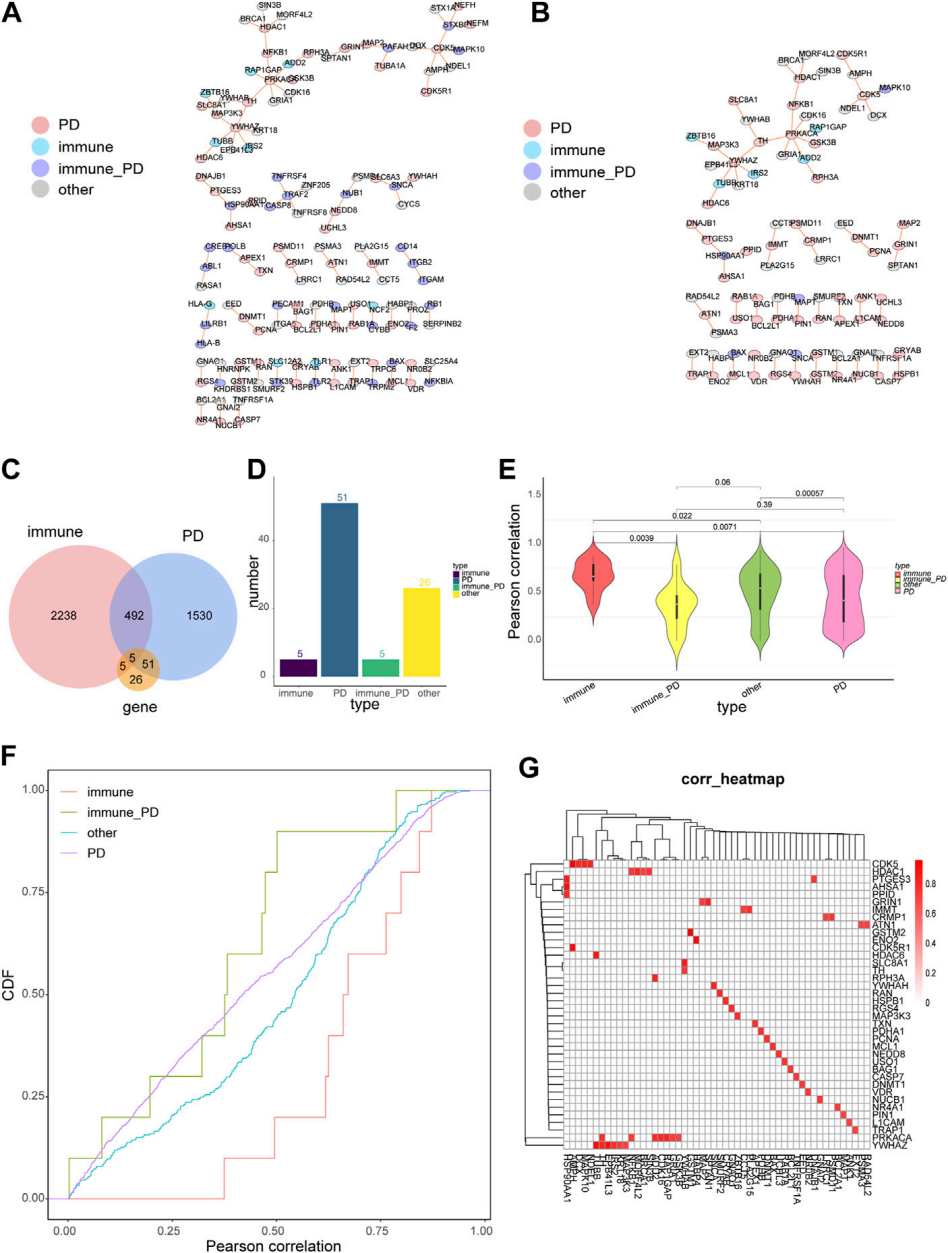
The properties of PD-directed neighbor co-expressed network (PDNC network). **(A)** The global PDNC network. **(B)** A sub-network was extracted from the PDNC network which comprised only PD genes and their linked genes. **(C)** A Venn diagram showed the intersections of PD-related genes, immune-related genes, and common network genes from the sub-network. **(D)** The histogram chart of the common genes included 5 immune-related genes, 51 PD-related genes, 5 immune-PD associated genes, and 26 other genes from the sub-network. **(E)** The violin plots of the Pearson correlations of the pairwise genes in the four gene categories. Wilcoxon rank-sum test was applied to compare the coexpression correlation coefficients between different gene groups. **(F)** The cumulative distribution curves of co-expression values (Pearson correlations) for diverse gene types. The vertical axis indicatd the degree of the expression correlation for each gene group. **(G)** The heatmap suggested the corrections between PD genes and their linked genes, Horizontal axis represents PD genes linked genes, vertical axis represents PD genes, and red squares indicated |Pearson coefficient value| > 0.7.

The core genes of PD, immune-PD genes, and their linked genes were extracted from the network of 3.1. Then, a network of these genes was constructed with 130 nodes and 91 nodes (Figure 2A, Supplementary Table S4). The genes whose core genes only were PD and the genes that were directly affected by PD were extracted to construct a network that including 87 nodes and 60 edges (Figure 2B, Supplementary Table S5). Totally five common genes were detected between 2078 PD genes, 2740 immune genes, and 87 core genes (Figure 2C). Furthermore, the number of genes in the four gene categories (PD, immune-PD, immune, and other genes) that the core genes only were PD of the PD gene interaction network were accounted (Figure 2D). Among the protein-interaction network, the number of immune genes was 5; the number of PD genes was 51; the number of immune-PD genes was 5; the number of other genes was 26 (Figure 2D). Moreover, there were significant differently expressed correlations between different gene groups, except immune-PD and PD genes (*p* = 0.39) (Figure 2E). In the PD genes and its linked genes, the correlation of immune genes, immune-PD genes and other genes was significant (Figure 2F). The correlation between PD genes and its linked genes was significant with |Pearson coefficient value| > 0.7 and FDR <0.05 (Figure 2G). Totally, the results showed that there were topological interactions and expression patterns among the correlations between PD- and immune-related genes.

### Module cluster analysis and validation of its classification power

**FIGURE 3:**
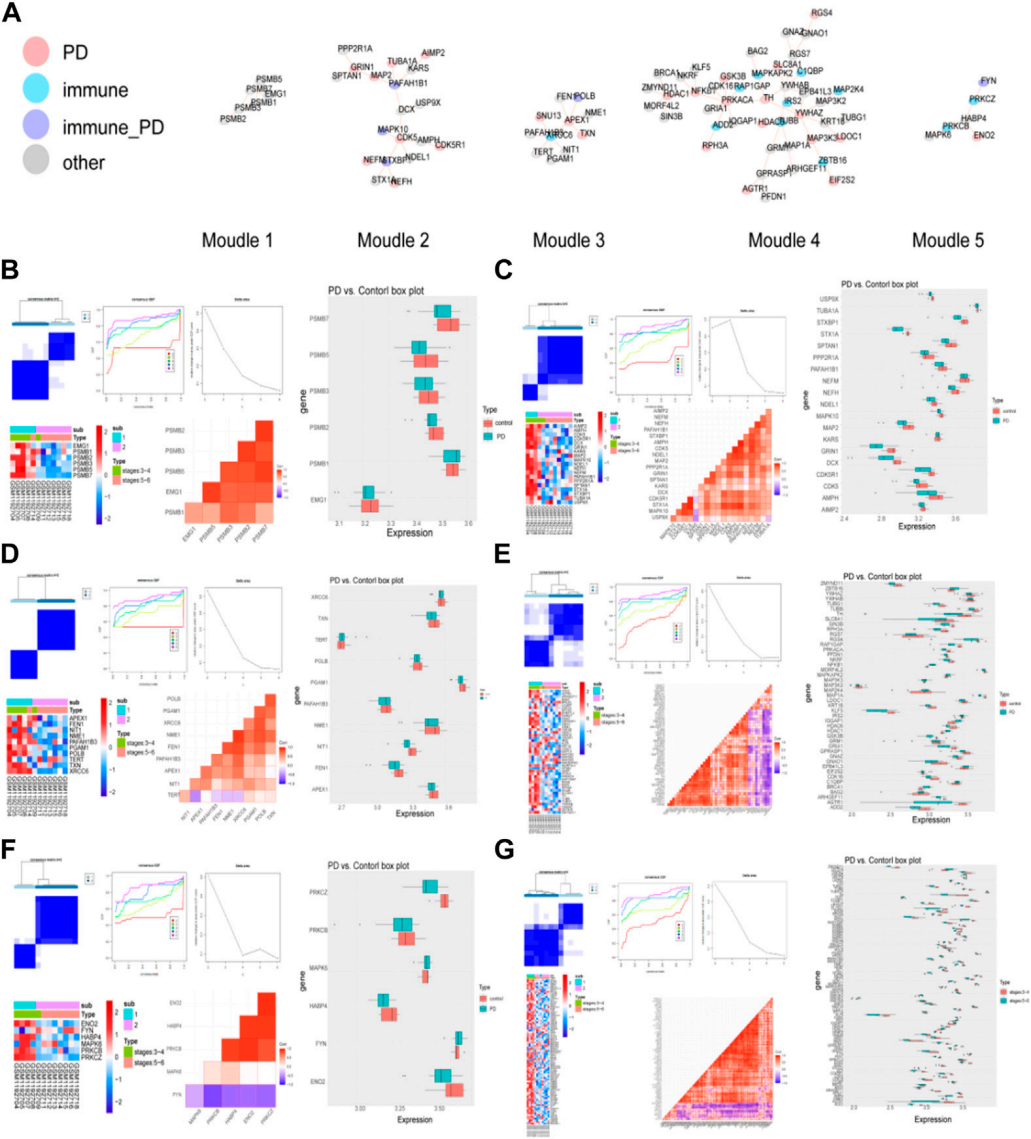
Detection of PD-related clusters and validation of their classification power. **(A)** Important clusters of modules were generated in the IPGDNC network. **(B**–**G)** The consensus cluster heatmap, cumulative distribution function (CDF) plot, delta area plot, gene expression heatmap, gene correlation heatmap, and gene expression box plot of modules including module 1 **(B)**, module 2 **(C)**, module 3 **(D)**, module 4 **(E)**, module 5 **(F)**, and the common genes **(G)**.

The modules with top 5 functional scores were selected (Figure 3A, Supplementary Table S6). In the all of 5 modules, 25 PD genes, 11 immune genes, 5 immune-PD genes, and 46 other genes were contained.

In these 6 modules, the genes were divided into 2 clusters when the K = 4, and the expression of these module genes were higher in cluster 1 (Figures 3B–G). In these modules, the expression levels of module genes except PSMB1, KARS, TERT, ZBTB16, NFKB1, MAPKAPK2, MAP3K3, MAP2K4, IRS2, IQGAP1, HDAC1, EPB41L3, C1QBP, BRCA1, BAG2, FYN, MAPK6, MAP3K2, and FNY in the PD group were higher than that in the control group.

**FIGURE 4:**
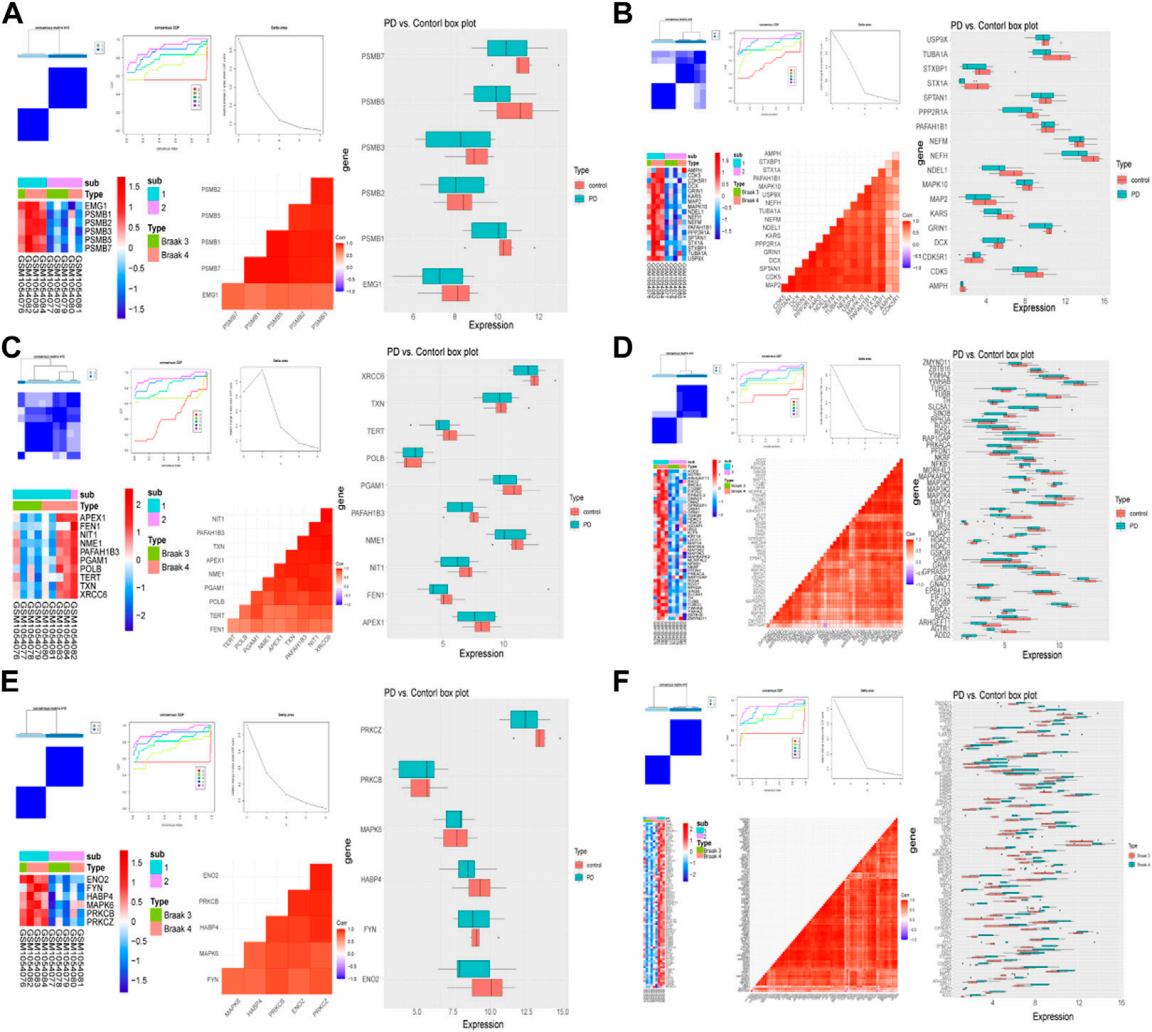
Validation of the classification power by GSE20292 data set. The consensus cluster heatmap, cumulative distribution function (CDF) plot, delta area plot, gene expression heatmap, gene correlation heatmap, and gene expression box plot of six modules including module 1 **(A)**, module 2 **(B)**, module 3 **(C)**, module 4 **(D)**, module 5 **(E)**, and common genes **(F)**.

The same method was used to validate the accuracy of the screened module genes in the external validation set GSE20292 data set (Figure 4). These genes have better representation and can screen out patients at different stages. These results demonstrated that the model genes could distinguish PD and control samples well.

### Functional enrichment analysis of modular genes

**FIGURE 5:**
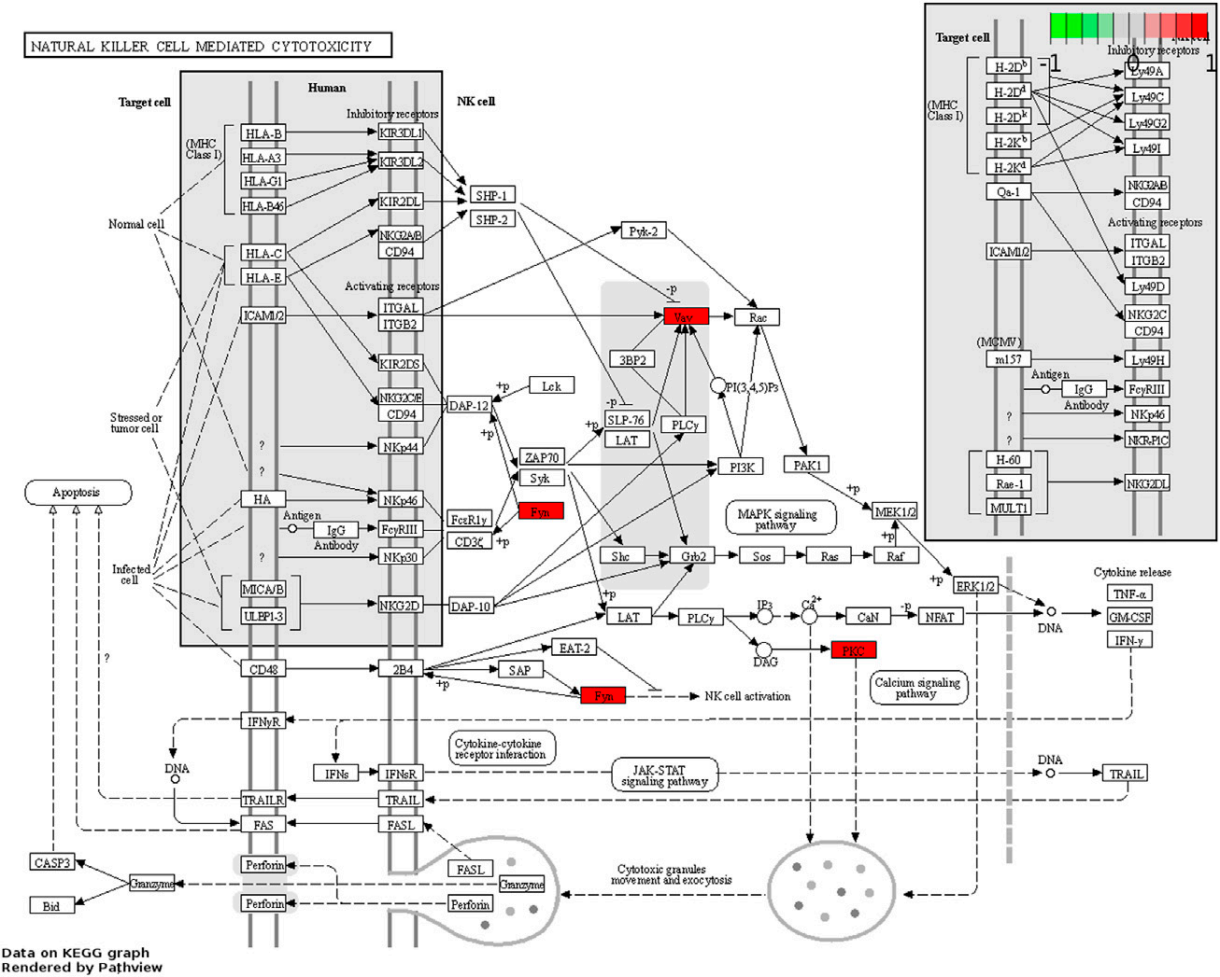
The map of natural killer (NK) cell mediated cytotoxicity signaling pathway. Red: up-regulation, grey: no significant difference.

Module 1 genes were enriched in 104 GO BPs (including 12 GO CCs, 7 GO MFs) and 8 KEGG pathways, and these GO terms and KEGG pathways were mainly related to various metabolic processes, complexes, and disease pathways (Supplementary Figures S1A, B). Module 2 genes were enriched in 303 GO BPs, 55 GO CCs, 12 GO MFs, and 7 pathways, and these GO terms and KEGG pathways were mainly related to various cell migration, protein binding, and disease pathways (Supplementary Figures S1A, B). Module 3 genes were enriched in 60 GO BPs, 17 GOs CC, 51 GO MFs, and 2 KEGG pathways, and these GO terms were mainly related to various telomere. The KEGG pathways were base excision repair and non-homologous end-ioining (Supplementary Figures S1A, B). Module 4 genes were enriched in 198 GO BPs, 35 GO CCs, 32 GO MFs, and 32 KEGG pathways, and these GO terms and KEGG pathways were mainly related to various protein binding, response to stimulus and disease pathways (Supplementary Figures S1A, B). Module 5 genes were enriched in 141 GO BPs, 30 GO CCs, 35 GO MFs, and 12 KEGG pathways, and these GO terms and KEGG pathways were mainly related to various response to stimulus and signaling pathways (Supplementary Figures S1A, B). All of the 5 module genes was enriched in 312 GO BPs, 80 GO CCs, 68 GO MFs, and 49 KEGG pathways, and these GO terms and KEGG pathways were mainly related to various protein binding and disease pathways (Supplementary Figures S1A, B). Additionally, the natural killer (NK) cell mediated cytotoxicity pathway was showed in Figure 5, Fyn, Vav and PKC were significantly enriched.

### Differential analysis of immune cell infiltration by module genes

**FIGURE 6:**
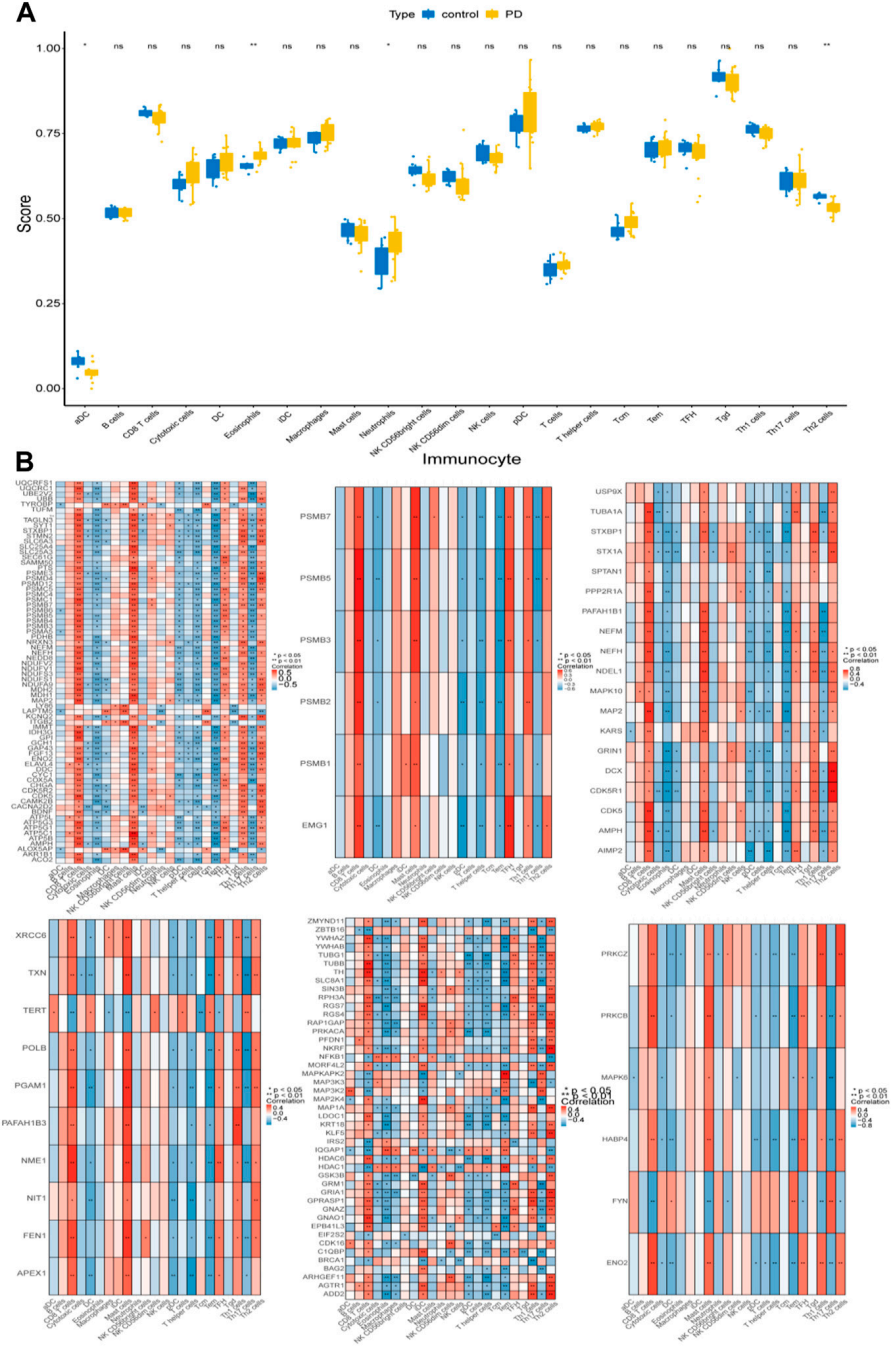
Immune cell infiltration analysis. **(A)** ssGSEA algorithm was performed to calculate the infiltration levels of 24 immune cell types in PD and normal samples. **(B)** The correlation heatmaps of 6 module genes with differential immune cells. * represented *p* < 0.05, ** indicated *p* < 0.01, ns represented no significant difference, red indicated positive correlation, blue indicated negative correlation.

To explore the differences in immune cell infiltration between the control and PD samples, the ssGSEA algorithm was performed. There were significant differences in aDC, eosinophils, neutrophils, and Th2 cells between the control and PD samples (Figure 6A). In addition, the genes of the 6 modules were significantly associated with these 4 differential immune cells (Figure 6B).

### Validation the expression of six module genes by qRT-PCR and western blot

**FIGURE 7:**
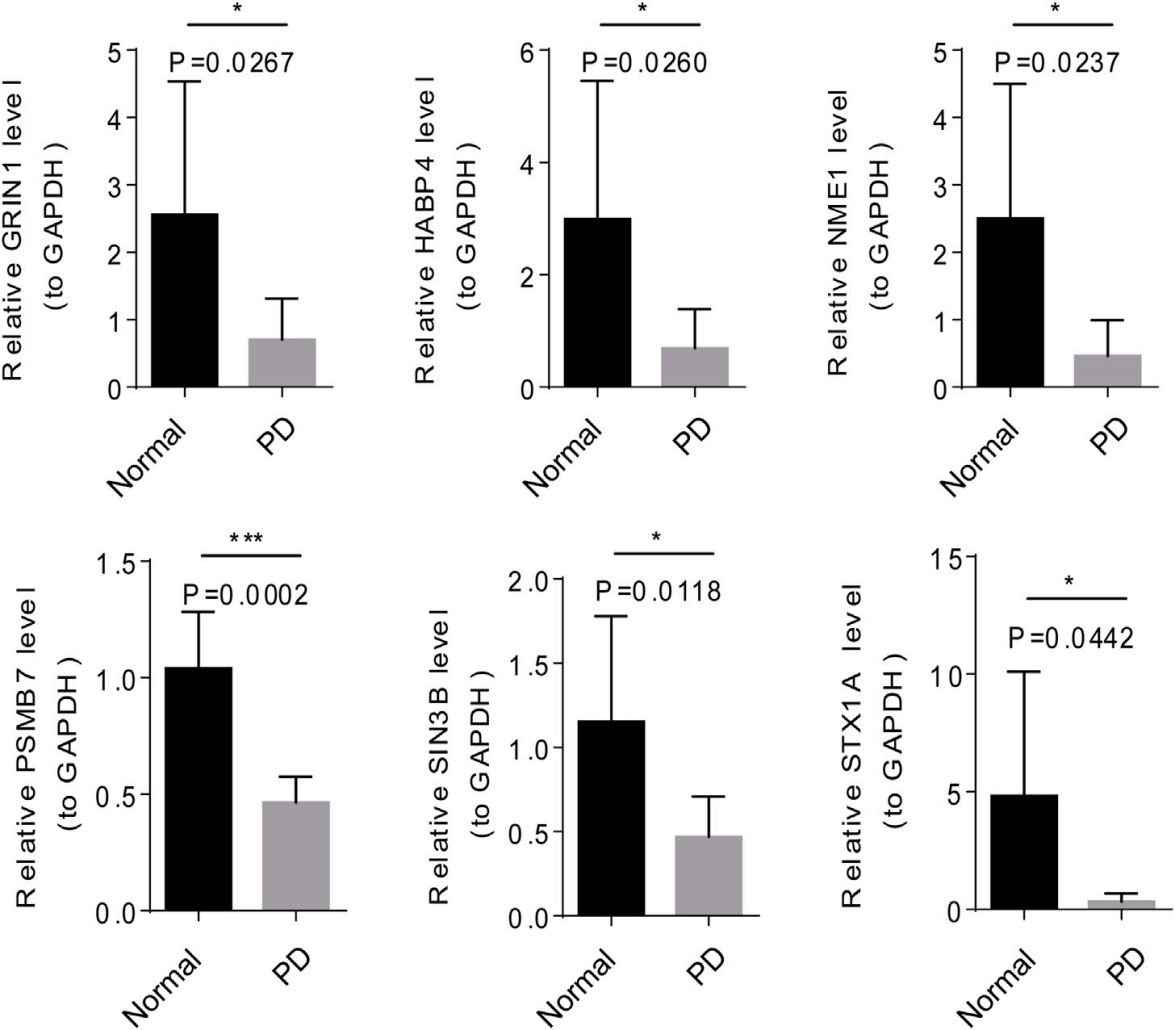
Verification of the mRNA expression of six modular genes by qRT-PCR and Western blot.

**FIGURE 8:**
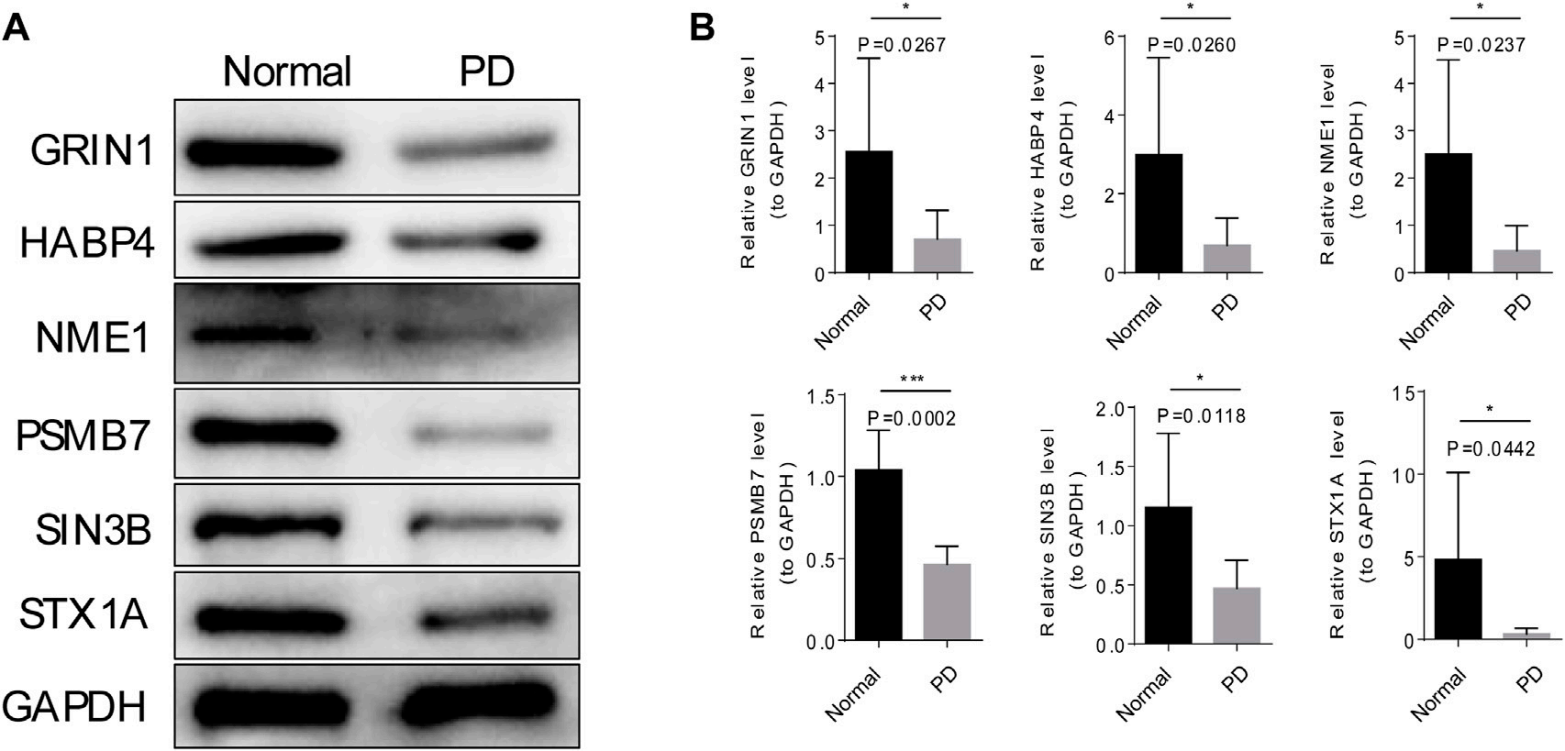
Verification of the protein expression of six modular genes by Western blot. **(A)** The grayscale values six of modular genes in PD and normal samples by Western blot. **(B)** The mages of six modular genes in PD and normal samples by Western blot.

The mRNA and protein expression of 6 module genes were significantly higher expressed in the normal samples than that in the PD samples (Figure 7 and Figure 8), the detailed statistical results for qRT-PCR and western blot were shown in Supplementary Table S7 and Supplementary Table S8, the original bar charts of western blot were shown in Supplementary Figure S2. These results confirm that these module genes could act as potential diagnostic markers for PD.

## Discussion

At present, the research on PD biomarkers has been gradually in-depth, but there is still no global study and recognition of some immune-related features. In this study, we explored the function and mechanism of immune-related genes in PD from a global perspective by integrating gene expression profiles from interaction networks and GEO databases.

The crosstalk between the peripheral immune system and neuroinflammation plays an important role in the pathogenesis of PD (Pajares et al., 2020). In our study, modular genes were mainly enriched in some stimulus-related categories and disease-related pathways. For example, the total module is enriched in Pathways of neurodegeneration - multiple diseases, Spinocerebellar ataxia, Prion disease, Alzheimer disease (AD), Huntington disease, PD, Long-term depression, and other disease pathways. Therefore, we speculated that the identified modules and genes played an important role in the development and progression of PD. It also further justified our typing of PD based on these genes. It has been suggested that genes involved in regulating substantia nigra development were enriched in RAC1+ NK cells and these cells showed increased brain infiltration in 1-methyl-4-phenyl-1,2,3,6-tetrahydropyridine (MPTP)-induced PD mice (Guan et al., 2022). Moreover, NK cells are also present in the brain parenchyma of mouse models of PD (Earls et al., 2020). NK cells can reduce synuclein burden *in vitro*, and systemic depletion of NK cells in a preclinical mouse model of PD results in increased pathological α-syn burden in numerous brain regions, including the striatum, SNpc, and brainstem (Peng et al., 2019). In the natural killer (NK) cell-mediated cytotoxicity pathway, Fyn, Vav, and PKC were significantly enriched. Among them, Fyn is a tyrosine phosphotransferase of Src family non-receptor kinases, which is mainly related to immune regulation, cell proliferation, and brain development (Guglietti et al., 2021). In previous studies, Fyn was confirmed to be a major upstream regulator of proinflammatory signaling pathway involving BDNF/TrkB, PKCδ, MAPK, AMPK, NF-κB, Nrf2, and NMDAR axis. Fyn is also being used as a potential signaling node for the development of novel anti-neuroinflammatory drug candidates for the treatment of PD and other related neurodegenerative diseases (Peng et al., 2019). For example, saracatinib, a non-selective Fyn inhibitor, has been tested in clinical trials to treat PD (Angelopoulou et al., 2021). The protein kinase C (PKC) family is a phospholipid-dependent serine/threonine kinase (Kishimoto et al., 1980). The protein kinase family consists of more than 15 subgroups and 500 kinases whose expression affects the progression of various diseases, including neurodegenerative diseases (Zisopoulou et al., 2013; Jha et al., 2015; Crawley et al., 2017). PKCα inhibits the expression of peroxisome proliferator-activated receptor C coactivator 1(PGC-1) by inducing miR-129–2 in neural tube defect (NTD) embryonic mouse models, and the overexpression of PGC-1 protects neurons from mitochondrial dysfunction under oxidative stress in PD (Mudò et al., 2012).

There is growing evidence linking the immune system to neuronal death and the pathogenesis of PD. Previous studies have shown that detection of immune cell components in the blood can identify the early stages of PD progression, leading to earlier detection and confirmation of PD (Farmen et al., 2021). Activated microglia (brain’s resident immune cells) correlate directly with the clinical and pathological severity of PD (Lanskey et al., 2018). Through immune infiltration analysis executed by the ssGSEA algorithm, we discovered that aDC and Th2 cells were significantly decreased in PD samples, and eosinophils and neutrophils cells were significantly upregulated in PD samples. But there is still a gap in how these cells play a role in the progression of cup-like lesions in Parkinson’s disease, however, this provided a basis and direction to further unravel the immune-related mechanisms of PD.

The top1 gene was selected from the 6 modules for qRT-PCR and Western blot validation. NME1 was a protein with serine/threonine specific protein kinase activity (Yu et al., 2021). NME1 has been shown to play an important role in neuronal growth by increasing mitochondrial respiration and preventing α-synuclein and LRRK2-induced degeneration. In PD treatment, NME1 can promote neurite growth in PD cell models and restore damaged mitochondrial respiration and cellular pathways (Anantha et al., 2022). GRIN1 (encoding NMDAR subunit n-methyl-D-aspartate 1) gene has been shown to be closely associated with neurodevelopmental disorders (Platzer et al., 1993), and its polymorphism has also been demonstrated as a potential biomarker for reducing the risk of PD in previous studies (Wu et al., 2010). The module genes such as the expression of NME1, SIN3B, HABP4, STX1A, SIN3B, HABP4, and STX1A could distinguish PD and normal samples, indicating these genes may become promising candidate genes for PD.

In conclusion, these results indicated strong correlations between immune- and PD-related genes not only in terms of network structures but also in expression patterns. According to the differential expression and functional enrichment analyses, some immune-related genes may have the potential as diagnostic and therapeutic biomarkers for PD. However, there still have two main limitations in this study. Firstly, this study was a retrospective study based on a public database with limited sample sizes. Second, the important genes and mechanisms in this study need further experimental studies to be validated. Altogether, we have revealed the association between immunity and PD through systematic network studies and bioinformatics approaches, providing a theoretical basis for further studies on the pathogenesis of PD and clinical therapeutic targets.

## Conclusion

In summary, all the results presented here indicate a strong association between immune and PD-related genes not only in network structure but also in expression patterns. After analyzing the expression patterns and functions of the genes in the five modules, we believe that these genes have potential as molecular diagnostic markers.

## Data Availability

The datasets presented in this study can be found in online repositories. The names of the repository/repositories and accession number(s) can be found in the article/Supplementary Material.
