# Supplementary material for: Identification of immune signatures in Parkinson’s disease based on co-expression networks
Source: Front Genet. 2023 Jan 17;14:1090382. doi: 10.3389/fgene.2023.1090382 (PMC9886886; doi:10.3389/fgene.2023.1090382)
Supplement: Supplementary file 3 [file Table9.DOCX]

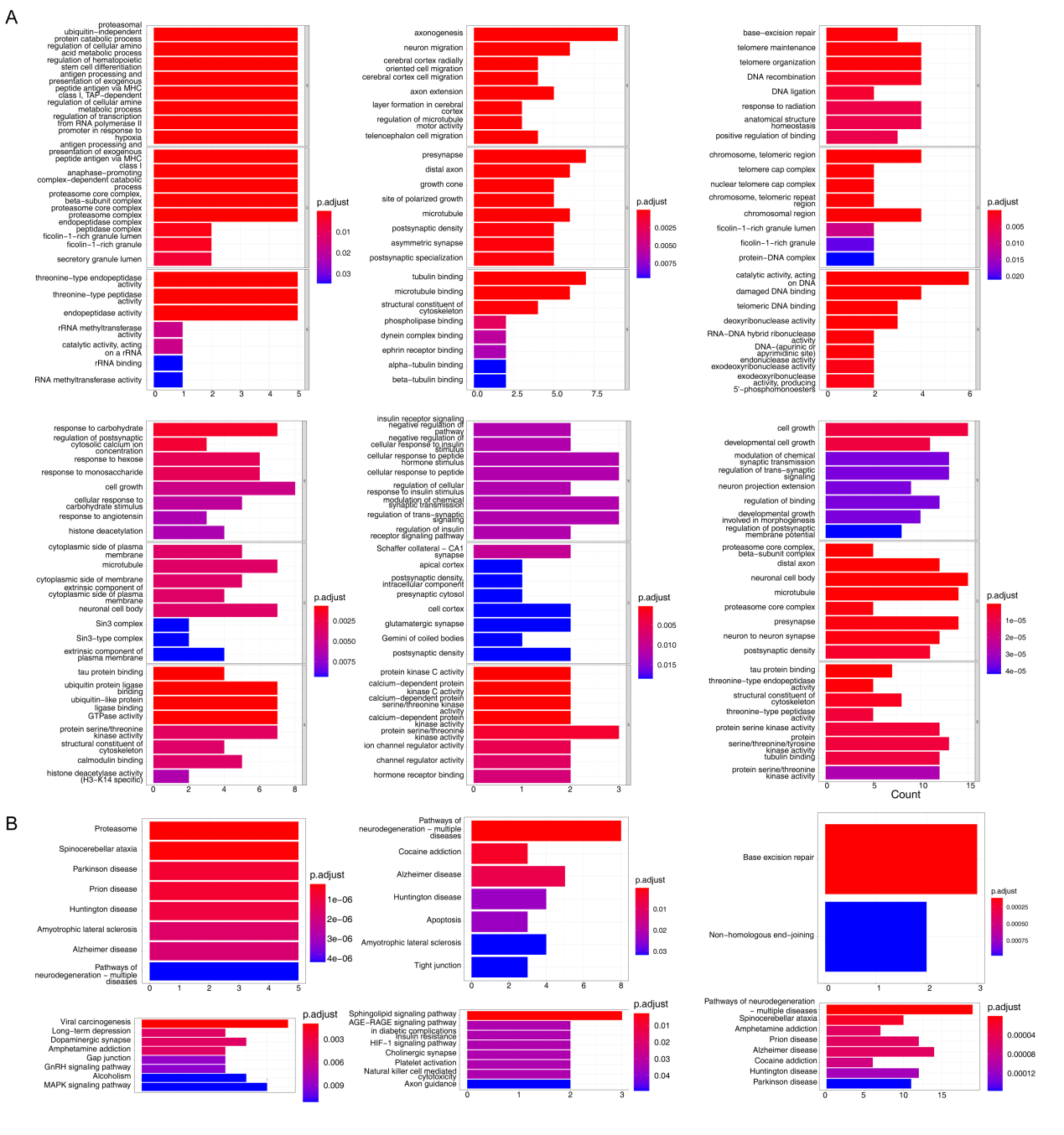


Supplementary Figure 1. Functional enrichment analysis of modular genes. (A) The GO enrichment analysis of different module genes. (B) KEGG pathway analysis of different module genes.


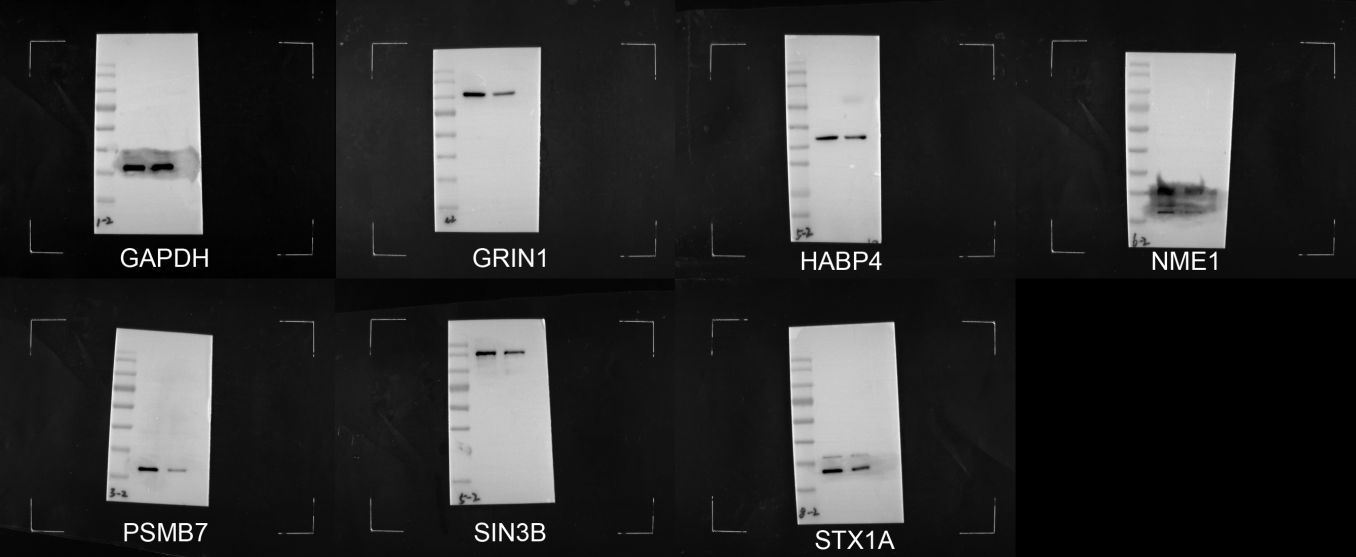


Supplementary Figure 2. The orginal bar charts of western blot.
